# miRNA‐34b/c regulates mucus secretion in RSV‐infected airway epithelial cells by targeting FGFR1

**DOI:** 10.1111/jcmm.16988

**Published:** 2021-10-12

**Authors:** Wenkai Li, Xizi Du, Yu Yang, Lin Yuan, Ming Yang, Ling Qin, Leyuan Wang, Kai Zhou, Yang Xiang, Xiangping Qu, Huijun Liu, Xiaoqun Qin, Gelei Xiao, Chi Liu

**Affiliations:** ^1^ Department of Pediatrics Hunan Provincial People’s Hospital The First Affiliated Hospital of Hunan Normal University Changsha China; ^2^ Centre for Asthma and Respiratory Disease School of Biomedical Sciences and Pharmacy Faculty of Health and Medicine University of Newcastle and Hunter Medical Research Institute Callaghan NSW Australia; ^3^ Department of Respiratory Medicine National Clinical Research Center for Respiratory Diseases Xiangya Hospital Central South University Changsha China; ^4^ Department of Physiology School of Basic Medicine Science Central South University Changsha China; ^5^ Department of Neurosurgery Xiangya Hospital Central South University Changsha China

**Keywords:** airway epithelial cells, FGFR1, miRNA, mucus secretion, respiratory syncytial virus

## Abstract

Respiratory syncytial virus (RSV) infection in airway epithelial cells is the main cause of bronchiolitis in children. Excessive mucus secretion is one of the primary symbols in RSV related lower respiratory tract infections (RSV‐related LRTI). However, the pathological processes of mucus hypersecretion in RSV‐infected airway epithelial cells remains unclear. The current study explores the involvement of miR‐34b/miR‐34c in mucus hypersecretion in RSV‐infected airway epithelial cells by targeting FGFR1. First, miR‐34b/miR‐34c and FGFR1 mRNA were quantified by qPCR in throat swab samples and cell lines, respectively. Then, the luciferase reporters’ assay was designed to verify the direct binding between FGFR1 and miR‐34b/miR‐34c. Finally, the involvement of AP‐1 signalling was assessed by western blot. This study identified that miR‐34b/miR‐34c was involved in c‐Jun‐regulated MUC5AC production by targeting FGFR1 in RSV‐infected airway epithelial cells. These results provide some useful insights into the molecular mechanisms of mucus hypersecretion which may also bring new potential strategies to improve mucus hypersecretion in RSV disease.

## 1 INTRODUCTION

Severe bronchiolitis is always caused by respiratory viruses during infancy, and respiratory syncytial virus (RSV) infection is the predominant reason. In hospitalized cases of infant bronchiolitis, the RSV infection rate is up to 50%–80%.^1^, ^2^ Compared with bronchiolitis induced by other common pathogens, such as rhinovirus (RV) and metapneumovirus, bronchiolitis caused by RSV is usually associated with prolonged hospital stay and intensive care,^3^ which is also characterized by young age and severe airway obstruction. Excessive mucus secretion is the critical reason for airway obstruction and aggravation of RSV infection.^4^ Moreover, severe cases of bronchiolitis in infancy are associated with a high risk of subsequent childhood asthma.^5^ Thus, infant bronchiolitis is assumed to be a key event in the secondary prevention strategy of asthma, as intervention subjects are infants and young children who have not developed asthma but have high‐risk signs of asthma (such as RSV infection). Add all this together, exploring the mechanism of abnormal mucus secretion after RSV infection has important implications for preventing the occurrence and development of severe bronchitis. Although the existing treatment is unable to satisfy the requirements of the secondary strategy, accumulating researches have verified that reducing the medical burden of bronchiolitis by cause‐based diagnosis and treatment has a deep and profound significance.^6^, ^7^ Therefore, deeper studies are needed to understand the specific pathological process of mucus secretion in early RSV infection.

miRNAs participate in gene expression which is involved in many biological processes, including cellular metabolism and immune responses.^8^ Of note, miRNAs in lungs have been verified to be an important regulatory factor of mucus secretion in airway epithelial cells.^9^, ^10^, ^11^ Our previous study has found that downregulated miRNA‐34b/c induces MUC5AC overexpression in severe RSV‐infected airway epithelia.^12^ However, in the process of mucus hypersecretion, the specific target and signalling pathway regulated by miR‐34b/miR‐34c is still obscure.

Bioinformatics analysis and miRNA binding site prediction revealed that Fibroblast growth factor receptor 1 (FGFR1) is the potential direct target in the process of mucin production regulated by miR‐34b/miR‐34c.^12^ FGFR1 belongs to the FGFR family, acts as a tyrosine kinase receptor to activate intracellular signalling pathways, including P13K, AKT, MAPK, etc.^13^ In airway epithelia, FGFR1 has been studied extensively which is involved in cell proliferation, stress response and epithelial‐mesenchymal transition (EMT), etc.^14^, ^15^ However, little is known about the influence of FGFR in epithelial mucin production. In this study, we first tested the expression of FGFR1 in airway epithelia after RSV infection in vitro and in vivo, respectively. Then, dual‐luciferase was used to confirm the direct binding between FGFR1and miR‐34b/miR‐34c. Finally, the FGFR1/AP‐1/MUC5AC pathway was detected to verify the involvement of FGFR1 in epithelial mucin production.

## 2 MATERIALS AND METHODS

### 2.1 Throat swab sample collection

All participants provided written informed consent which was approved by the No.2020KT‐52 of Central South University Research Ethics Committee. Children cases of RSV infection and healthy controls were recruited at Hunan Provincial People's Hospital between September 2020 and April 2021. Eligible patients (RSV patients group) were aged under 18 years and had a clinical diagnosis of RSV infection, bronchiolitis or asthmatic pneumonia. Patients who had pneumonia, acute heart failure within a month were excluded. Healthy controls were aged under 18 years without respiratory disease. Throat swab samples were collected for extracting RNAs and qPCR analysis using BIOG RNA Swab Kit (BAIDAI).^16^

### 2.2 Cell culture, treatment and RSV infection

Normal primary human bronchial epithelial cells (HBECs) and A549 cells were purchased and cultured following the previous literature.^12^, ^17^ Purified RSV‐A2 (MOI = 3) was incubated with HBECs or A549 cells for 24 h.

### 2.3 miRNA mimics and inhibitor treatment

Mimics of miR‐34b/miR‐34c and its negative control (RiboBIO) were added into HBECs or A549 cells with transfection reagent Lipofectamine 3000 (Invitrogen) for 48 h.^18^, ^19^ After transfection, cells were stimulated with RSV‐A2.^12^ To inhibit FGFR1, HBECs were incubated with 1 μmol/L PD173074 or medium for 24 h before RSV infection and miRNA mimics transfection.

### 2.4 Screening and analysis of public RNA profiles from RSV infected patients

Transcriptional profiles of RSV‐infected patients and controls were screened and selected from the *GEO* database. The screened target datasets were included according to the following criteria: (1) whole blood sample, nasal/throat swab sample, bronchial lavage fluid or induced sputum from human; (2) RNA‐seq; (3) the presence of RSV was confirmed by quantitative real‐time PCR. Profile graph function from the GEO2R program was used to obtain FGFR1 expression value and expression profile graph in different groups. The information of these datasets is described in Table 1.

**TABLE 1:** Characteristics of included public datasets

Dataset	Spices	Sample type	Group and details		
GSE105450	Human	Whole blood		RSV patients, *n* = 56	Healthy controls, *n* = 38
			Age	4.785 ± 0.5206	7.586 ± 0.7239
			Gender (Female/male)	24/32	13/25
GSE97742	Human	Nasopharyngeal swaps		Acute RSV patients, *n* = 38	Discharge RSV patients, *n* = 38
			Age	7.450 ± 0.7688	7.350 ± 0.7267
			Gender (Female/male)	11/27	11/27
GSE117827	Human	Nasopharyngeal swaps		RSV infection patients, *n* = 6	Asymptomatic controls, *n* = 6
			Age	5.600 ± 0.5416	32.33 ± 8.531
			Gender (Female/male)	4/2	0/6

### 2.5 RNA extraction, qRT‐PCR, miRNA qRT‐PCR

Total RNA was extracted from HBECs with RNAiso Plus (Takara). All throat swab samples were pretreated in DNA/RNA Shield(Zymo Research)and mRNA was extracted using BIOG RNA Swab Kit (BAIDAI). The PrimeScript RT Master Mix Kit (Takara) was employed to access the mRNA expression, and the miRcute miRNA cDNA kit (Tiangen Biotech) was used to detect miRNA expression. qPCR procedure was executed by a CFX96 Touch™ Deep Well Real‐Time PCR Detection System (Bio‐RAD) with TB Green P remix Ex Taq (Takara). The primers are listed in Table S1. β‐actin and U6 serve as internal controls, respectively.

### 2.6 Western blot

The steps of western blot were described in previous publications.^20^ Briefly, cells are lysed by RIPA lysis buffer and protein were collected. Total protein was separated by 10% SDS‐PAGE and transferred to polyvinylidene fluoride (PVDF) membrane. Then, 5% bovine serum albumin (BSA) was used to block non‐specific sites. After that, membranes were incubated with primary antibodies overnight at 4℃ and incubated with secondary antibody the next day. The primary antibodies are listed as follows: c‐Jun (Santa Cruz, sc‐74543), FGFR1 (Abcam, ab824), β‐actin(Santa Cruz, sc‐84322) and phosphorylated c‐Jun (Santa Cruz, sc‐822).

### 2.7 Dual‐luciferase reporter assay

The binding regions of FGFR1‐ miR‐34b and FGFR1‐miR‐34c were predicted using the *Targetscan* databases (http://www.targescan.org/). The sequence of FGFR1 3′ untranslated regions (3′ UTR) was amplified by PCR and cloned into GV272 vector (containing reporter gene sequence) to construct FGFR1 wild‐type (3′UTR‐WT) and mutant (3′UTR‐MUT) dual‐luciferase reporter plasmids, respectively. Then, using Lipofectamine 3000 (Invitrogen), the luciferase reporters (including miR‐NC, miR‐34b mimic or miR‐34c mimic) were cotransfected into HBECs or A549 cells, respectively. The luciferase intensities were determined with the Dual‐Luciferase Reporter Gene Assay Kit (Beyotime) by normalizing the firefly luminescence to Renilla luminescence.

### 2.8 Cell Counting Kit‐8 assays

To accessed cell viability, HBECs were cultured and grown to a density of 70% in a 96‐wells plate. After being treated by miR mimic or miR NC, cell viability assays were operated following the CCK‐8 kit protocol (Dojindo). The cell density at 450 nm was determined using a microplate reader (Bio‐rad).

### 2.9 Statistical analysis

The differential expression between the RSV infection group and the control group in each database was presented as mean ± SD, *p*‐value < 0.05, analysed by unpaired *t*‐test. Other data were analysed with GraphPad Version 7 from minimum of three independent experiments, presented as mean ± SEM, analysed by one‐way analysis of variance (ANOVA) or unpaired *t*‐test. *p*‐value < 0.05 were considered differences significant.

## 3 RESULTS

### 3.1 The protein level of FGFR1 was upregulated in RSV‐infected airway epithelial cells

**FIGURE 1:**
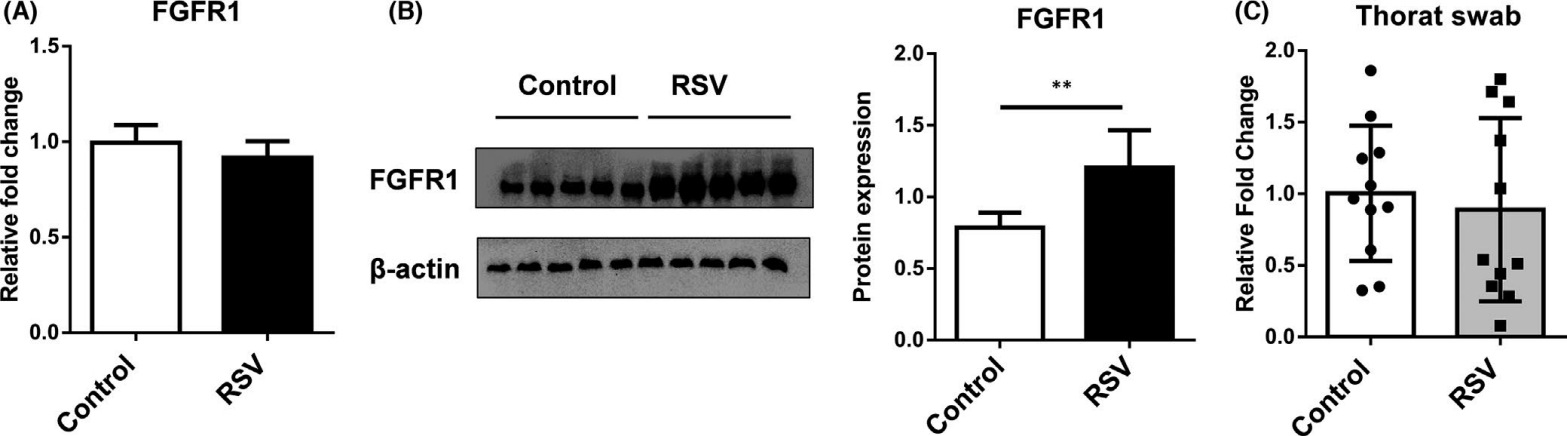
The protein level of FGFR1 in airway epithelia increase significantly after RSV infection. (A) qPCR was performed to examine FGFR1 mRNA level in RSV‐infected HBECs. (B) Western Blot was performed to examine FGFR1 protein level in RSV‐infected HBECs. (C) mRNA expression of FGFR1 in throat swab samples was analysed from RSV patients (RSV, *n* = 11) and healthy controls (Control, *n* = 11). **p < 0.05

To verify the involvement of FGFR1 in airway epithelia after RSV infection, the mRNA and protein level of FGFR1 were analysed in RSV‐infected HBECs. As shown in Figure 1A, there is no significant change of FGFR1 mRNA expression between RSV‐infected HBECs and controls. However, FGFR1 protein expression was significantly higher in RSV‐infected HBECs than controls (Figure 1B). Moreover, the mRNA level of FGFR1 was also analysed in throat swab samples from 11 healthy controls and 11 RSV patients. Consistent with the in vitro results, no significant difference has been found between the two enrolled clusters (Figure 1C). These results demonstrated that the protein level of FGFR1 in airway epithelia was upregulated after RSV infection, but no significant difference was detected in the mRNA level of FGFR1.

### 3.2 FGFR1 expression was analysed by RNA profiling in RSV‐infected patient datasets

**FIGURE 2:**
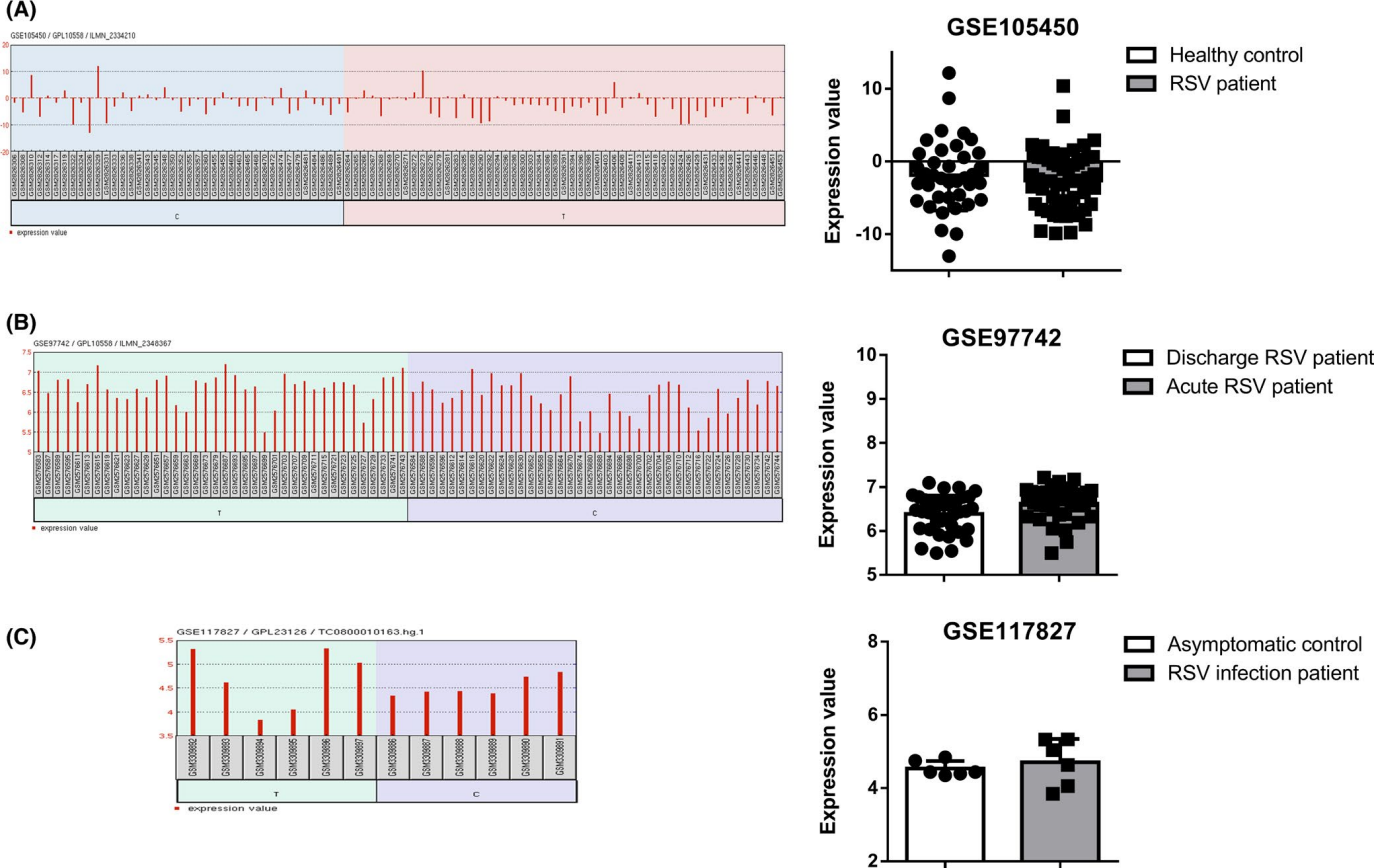
Fibroblast growth factor receptor 1 expression in RSV‐infected patients and healthy controls in public RNA profiling datasets. (A) FGFR1 expression profile graph in GSE105450 and differential expression analysis between RSV patients and healthy controls. (B) FGFR1 expression profile graph in GSE97742 and differential expression analysis between acute RSV patients and discharge RSV patients. (C) FGFR1 expression profile graph in GSE117827 and differential expression analysis between RSV infection patients and asymptomatic controls

To further confirm the expression mode of FGFR1 after RSV infection, we screened RNA profiling subjecting of RSV‐infected patients and healthy controls from public databases. Three datasets (GSE105450, GSE97742, GSE117827) were finally included in this study according to previous criteria. Characteristics of these three datasets was shown in Table 1. We analysed the differential expressed genes and the RNA‐seq value of FGFR1 in each dataset which are shown in Figure 2. Consistent with expectations, there is no significant difference in FGFR1 mRNA expression between the RSV infection group and the control group. Thus, RSV infection does not influence the mRNA expression of FGFR1 in vivo.

### 3.3 miR‐34b/miR‐34c inhibited MUC5AC overexpression in RSV‐infected airway epithelial cells

**FIGURE 3:**
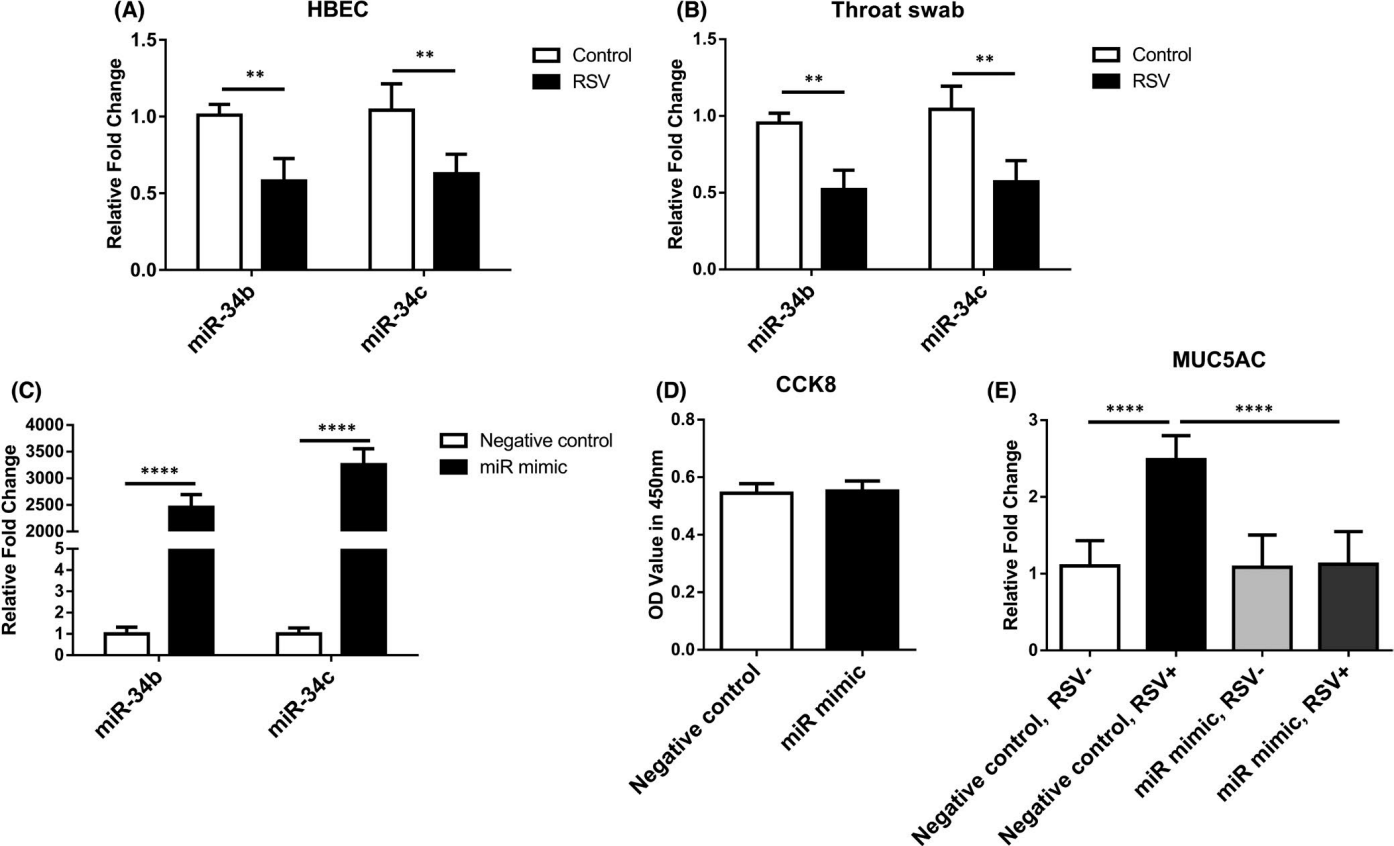
miR‐34b/miR‐34c overexpression inhibited MUC5AC expression in RSV‐infected airway epithelial cells. (A) Expression of miR‐34b and miR‐34c in RSV‐infected HBECs (B) Expression of miR‐34b and miR‐34c in throat swab samples from RSV patients (*n* = 11) and healthy controls (*n* = 11). (C) HBECs was transfected with miR‐34b or miR‐34c mimic, qPCR validate the expression of miR‐34b/miR‐34c after transfection. (D) HBECs treated with miR‐34b mimic and miR‐34c mimics (miR mimic) or negative control mimic (Negative control) were subject to CCK8 assay. (E) The mRNA expression of MUC5AC was analysed by qPCR after the transfection of miR‐34b/miR‐34c mimics in RSV‐infected (RSV+) or control (RSV‐) HBECs. **p < 0.05, ***p < 0.01

Next, we investigated the expression changes and the influence of miR‐34b/miR‐34c in airway epithelia during RSV infection. Our previous research found the RSV infection decreased the expression of miR‐34b/miR‐34c in airway epithelia which could further induce MUC5AC overexpression.^12^ Here, the level of miR‐34b/miR‐34c after RSV infection was further analysed in HBECs and throat swab samples, respectively. As is shown in Figure 3A,B, not only in HBECs but also in throat samples, the expression of miR‐34b/miR‐34c from RSV‐infected HBECs were at a significantly lower level than the corresponding control HBECs. The transient transfection of miR‐34b/34c mimics obviously elevated the level of miR‐34b/miR‐34c in HBECs (Figure 3C). In addition, cell viability assay showed that miR‐34b/ miR‐34c mimics have no effect on cell viability (Figure 3D). Moreover, miR‐34b/miR‐34c overexpression blocked MUC5AC expression significantly in HBECs after RSV infection (Figure 3E).

### 3.4 miR‐34b/ miR‐34c directly target FGFR1 in HBECs

**FIGURE 4:**
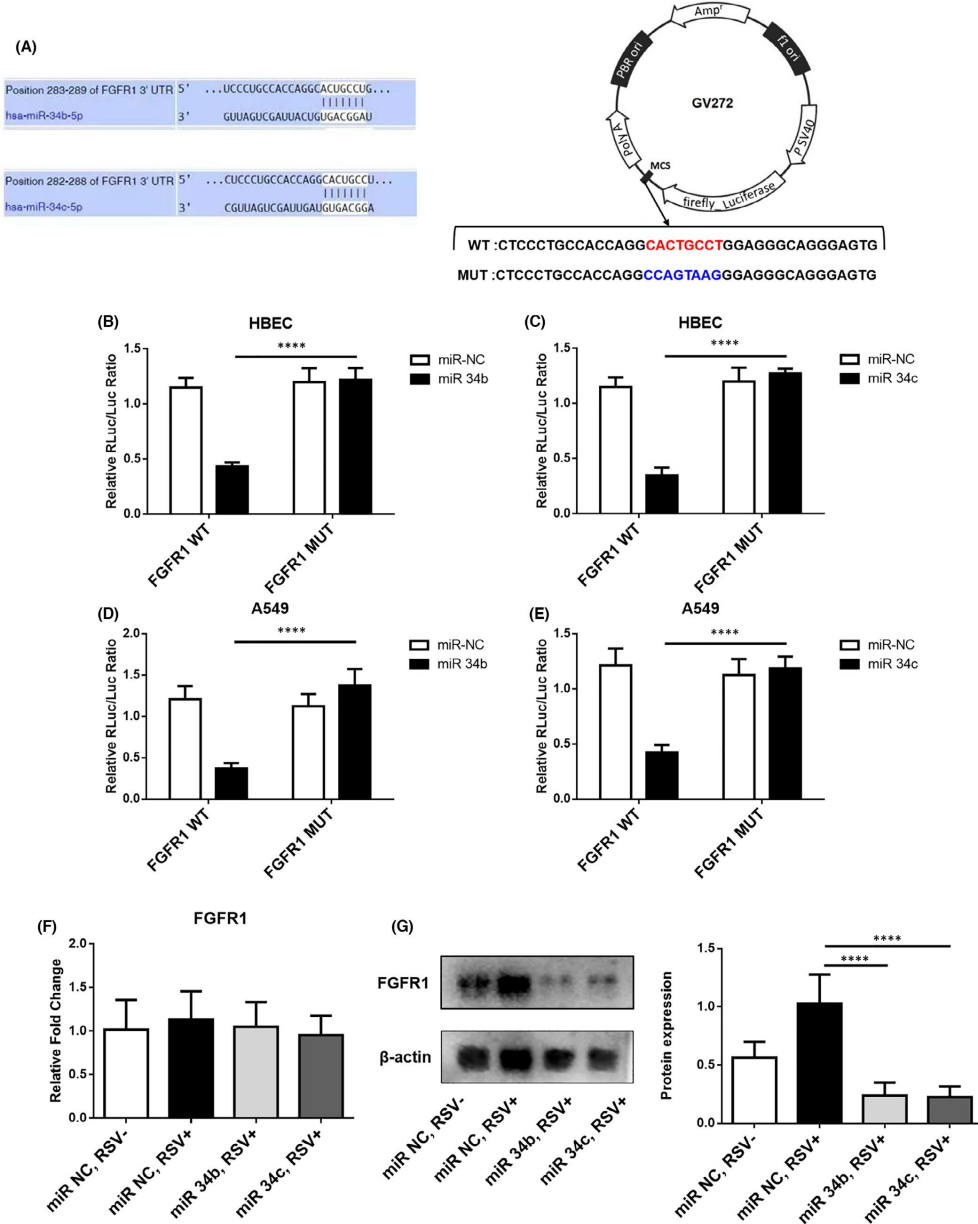
Fibroblast growth factor receptor 1 is identified as a direct target of miR‐34b/miR‐34c in airway epithelial cells. (A) The predicted miR‐34b/miR‐34c complementary sequence with the 3′‐UTR region of FGFR1, as well as the mutant containing altered nucleotides in the 3′‐UTR of FGFR1. (B) Luciferase activity assay was operated after transfection with FGFR1‐WT or FGFR1‐MUT reporter and miR‐NC or miR‐34b in HBECs. (C) Luciferase activity assay was operated after transfection with FGFR1‐WT or FGFR1‐MUT reporter and miR‐NC or miR‐34c in HBECs. (D) Luciferase activity assay was operated after transfected cells with FGFR1‐WT or FGFR1‐MUT reporter and miR‐NC or miR‐34b in A549 cells. (E) Luciferase activity assay was operated after transfected with FGFR1‐WT or FGFR1‐MUT reporter and miR‐NC or miR‐34c in A549 cells. (F) qPCR was used to analyse the mRNA expression of FGFR1 in RSV‐infected HBECs in the presence of miR‐34b/miR‐34c overexpression. (G) Western blotting was performed to examine the effect of miR‐34b/ miR‐34c overexpression on the protein level of FGFR1 in RSV‐infected HBECs. ****p < 0.001

Using the algorithm in *TargetScan*, we further assessed the possible targets of miR‐34b/ miR‐34c.^21^ The predicted result identified that 3′‐UTR of FGFR1 has complementarity regions with miR‐34b/ miR‐34c, respectively (Figure 4A), which was taken up for further validation.

Then, HBECs and A549, which represented the major target cells of RSV infection, were used as in vitro models.^22^, ^23^ The interaction of this FGFR1 with miR‐34b/miR‐34c was examined by the luciferase reporter gene containing the binding site of WT or MUT FGFR1. Compared with FGFR1 MUT, the luciferase density driven by FGFR1 WT and miR‐34b/miR‐34c mimics was decreased conspicuously both in A549 cells and HBECs (Figure 4B–E). Then, the effects of miR‐34b/miR‐34c on the expression of FGFR1 were further verified, which revealed that miR‐34b/miR‐34c mimics induced the suppression of FGFR1 (Figure 4G). These results suggest that the binding of FGFR1 3′‐UTR ‐ miR‐34b/c inhibited the expression of FGFR1. In addition, the overexpression of miR‐34b/miR‐34c significantly reduced the protein expression of FGFR1, but not the mRNA expression (Figure 4F). Thus, RSV infection in airway epithelia only upregulated the protein expression of FGFR1. The above results suggested that the regulation of FGFR1 by miR‐34b/miR‐34c may inhibit its post‐transcriptional translation by loosely binding to its 3’UTR region.

### 3.5 miR‐34b/ miR‐34c induced MUC5AC overexpression through AP‐1 signalling in RSV‐infected HBECs by targeting FGFR1

**FIGURE 5:**
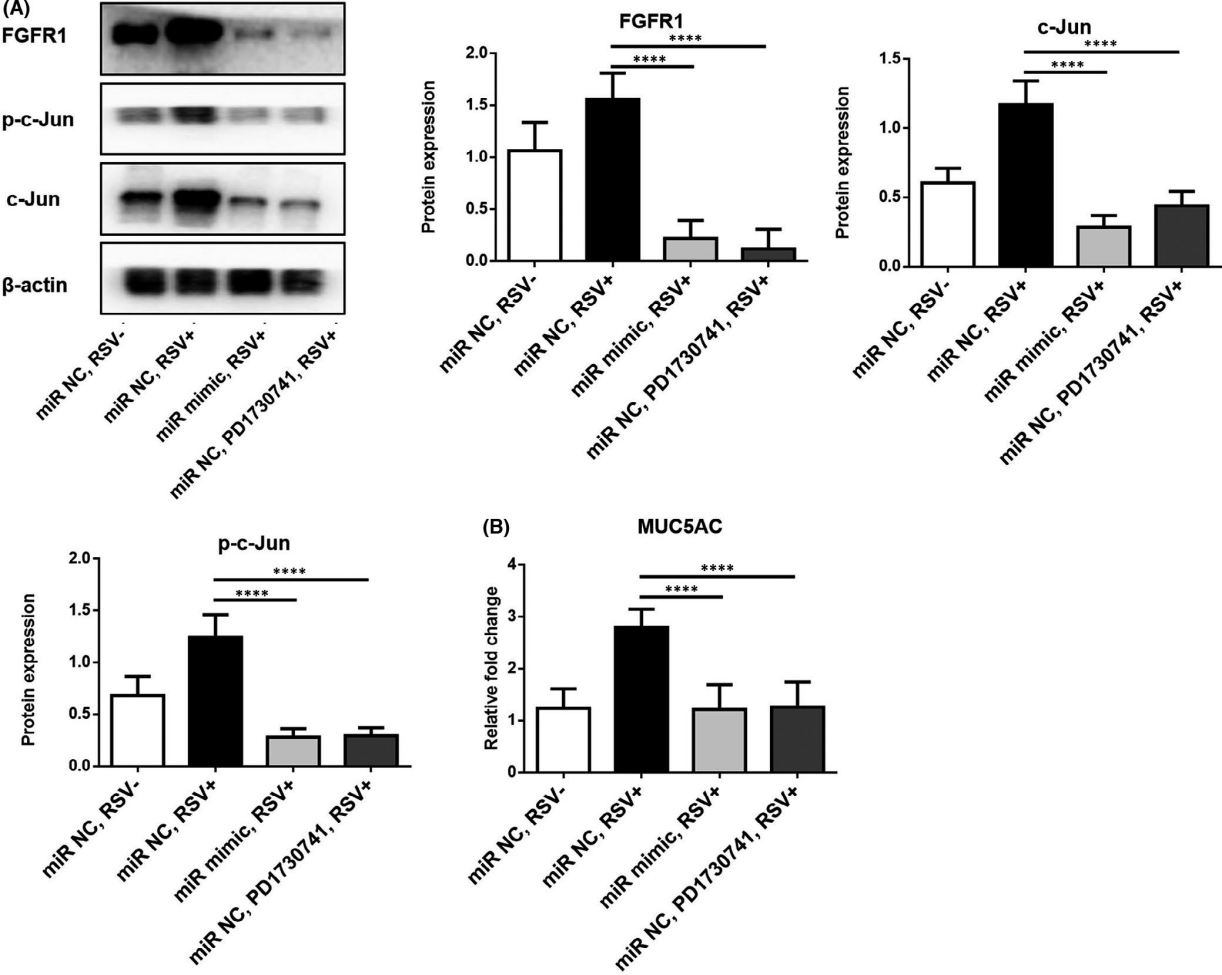
Respiratory syncytial virus mediated activation of c‐Jun/MUC5AC signalling was blocked by FGFR1 inhibition. (A) Western blotting was used to assess the effects of miR‐34b/miR‐34c mimics or FGFR1 inhibitor (PD1730741) on the protein levels of p‐c‐Jun and c‐Jun in RSV‐infected HBECs. (B) qPCR was used to assess the effects of miR‐34b/miR‐34c mimics or PD1730741 on the mRNA levels of MUC5AC in RSV‐infected HBECs. ****p < 0.001

It had been proved that through activating c‐Jun, a part of transcription factor AP‐1, the decreased miR‐34b/miR‐34c contribute to RSV‐induced abnormal secretion of mucin.^12^ In this study, we further explored whether miR‐34b/miR‐34c could exert its effects on c‐Jun activation by targeting FGFR1. The results demonstrated that RSV infection significantly increased FGFR1 and c‐Jun expression, as well as phosphorylated c‐Jun. This effect was eliminated by delivering exogenous miR‐34b/miR‐34c. Meanwhile, FGFR1 inhibitor PD1730741 or miR‐34b/miR‐34c mimics inhibited c‐Jun phosphorylation with conspicuous reversal of FGFR1 expression (Figure 5A). PD1730741 also inhibited MUC5AC overexpression in HBECs after RSV infection (Figure 5B). These results suggested that the RSV infection upregulated FGFR1‐activated AP‐1 which subsequently induced MUC5AC overexpression in airway epithelial cells.

## 4 DISCUSSION

Respiratory syncytial virus infection is the most common respiratory system infection in the period of infants and young children. The severity of RSV infection varies from mild symptoms of the common cold to airway obstruction, even hypoxia and pneumonia.^24^ Mucus hypersecretion is one of the main hallmarks of RSV infection. Excessive mucus secretion with cell debris will obstruct the bronchioles which is a critical reason for the aggravation of RSV infection.^25^, ^26^ Moreover, excessive mucus with pro‐inflammatory cell infiltration constitutes an inducement for airway hyperresponsiveness (AHR),^27^, ^28^, ^29^, ^30^, ^31^ which also related to the occurrence of asthma in later childhood. Thus, therapeutic interventions of mucus hypersecretion have profound significance in preventing RSV disease and asthma in children. However, about 75% of RSV‐infected children in hospitals do not have risk factors such as preterm birth or comorbidities.^32^ Studies have demonstrated that there are some other potential host factors that are involved in the regulation of RSV infection.

In recent years, the regulation of respiratory virus infections by miRNAs has been extensively studied. Our previous study also found that miRNA‐34b/c contributed to the RSV‐induced mucin production in airway epithelial cells through the functional modification of c‐Jun, the AP‐1 subunit.^12^ However, c‐Jun was not the direct‐targeted gene of miRNA‐34b/c. In this study, we confirmed the decreased expression of miR‐34b/miR‐34c after RSV infection from throat swab samples and airway epithelial cells, respectively. Then, the direct binding between FGFR1 and miR‐34b/miR‐34c was verified through dual‐luciferase experiment verification in this study. Also, the involvement of FGFR1 in MUC5AC overexpression by regulating c‐Jun was validated in RSV‐infected airway epithelial cells.

Existed literatures have demonstrated the interaction between FGFR1 and c‐Jun.^33^, ^34^, ^35^, ^36^, ^37^ FGFR1 shows enriched expression in head and neck squamous cell carcinoma (HNSCC) which can activate ERK1/2, p38 and c‐Jun. FGFR1 inhibitors blocked the AP‐1 pathway which can further induce mesenchymal‐epithelial transition (MET).^33^ In addition, activation of the FGFR/FRS2α signalling pathway in liposarcoma is positively correlated with the overexpression of c‐Jun.^34^, ^35^ Moreover, in bone marrow stromal cells and retinal pigment epithelial cells, FGFR1 regulates c‐Jun and further influences the signal mechanism of AP‐1 activation.^36^, ^37^ Consistent with the previous findings, our results further verified the regulation of FGFR1 on AP‐1 signalling in airway epithelial cells following RSV infection.

It is known that the downregulation of miR‐34b/miR‐34c was engaged in MUC5AC overexpression in RSV‐infected airway epithelial cells.^12^, ^38^ Here, we further verified that miR‐34b/miR‐34c inhibited the expression of FGFR1. Intriguingly, our results demonstrated that the negative regulation of miR‐34b/miR‐34c on FGFR1 only affects the protein expression of FGFR1, but not the mRNA expression. These results suggested that the post‐transcriptional regulation of FGFR1 by miR‐34b/miR‐34c is achieved by regulating the process of protein translation. This is partly due to the incomplete complementarity and loose combination of FGFR1 3'UTR with miR‐34b/miR‐34c.

Although our research confirmed the key role of FGFR1 in epithelial mucus hypersecretion after RSV infection which is modulated by miR‐34b/miR‐34c, there are still some limitations. The first one is that the inhibitory level of miR‐34b/miR‐34c on c‐Jun/MUC5AC activation should be detected through the FGFR1 overexpression plasmid. Besides, xenograft mouse models or organoids should be applied to study the function of FGFR1 and miRNA‐34b/c after RSV infection.

In conclusion, our study validated that miR‐34b and miR‐34c regulate the overexpression of MUC5AC in RSV‐infected airway epithelial cells by targeting FGFR1, which further induce mucus hypersecretion. This study offers some novel perceptions of the mechanisms of RSV‐induced mucus secretion which may also bring novel strategies to treat mucus hypersecretion and RSV infection effectively.

## CONFLICT OF INTEREST

The authors confirm that there are no conflicts of interest.

## AUTHOR CONTRIBUTIONS

**Wenkai Li:** Data curation (equal); Formal analysis (equal); Writing‐original draft (equal). **Xizi Du:** Data curation (equal); Formal analysis (equal); Writing‐original draft (equal). **Yu Yang:** Investigation (equal). **Lin Yuan:** Investigation (equal). **Ming Yang:** Funding acquisition (equal); Writing‐review & editing (equal). **Ling Qin:** Investigation (equal). **Leyuan Wang:** Data curation (equal). **Kai Zhou:** Data curation (equal). **Yang Xiang:** Funding acquisition (equal). **Xiangping Qu:** Funding acquisition (equal). **Huijun Liu:** Methodology (equal). **Xiaoqun Qin:** Funding acquisition (equal). **Gelei Xiao:** Funding acquisition (equal); Supervision (equal); Writing‐review & editing (equal). **Chi Liu:** Data curation (equal); Formal analysis (equal); Funding acquisition (equal); Writing‐original draft (equal).

## Supporting information

Table S1

## Data Availability

The data that support the findings of this study are available in the National Center for Biotechnology Information (NCBI) Gene Expression Omnibus (GEO) database with accession code: GSE105450 (https://www.ncbi.nlm.nih.gov/geo/query/acc.cgi?acc=GSE105450), GSE97742 (https://www.ncbi.nlm.nih.gov/geo/query/acc.cgi?acc=GSE97742), GSE117827 (https://www.ncbi.nlm.nih.gov/geo/query/acc.cgi?acc=GSE117827).
