# Supplementary material for: miRNA‐34b/c regulates mucus secretion in RSV‐infected airway epithelial cells by targeting FGFR1
Source: J Cell Mol Med. 2021 Oct 12;25(22):10565–74. doi: 10.1111/jcmm.16988 (PMC8581336; doi:10.1111/jcmm.16988)
Supplement: Supplementary file 1 — Table S1 [file JCMM-25-10565-s001.docx]

**Supplementary Table 1**. Primer sequence of genes for qPCR.

| Gene | Primer sequence |
| --- | --- |
| FGFR1(human) | Forward: 5’- GGTTGACCGTTCTGGAAGC -3’ |
|  | Reverse: 5’- GCCCCGGTGCAGTAGATA -3’ |
| MUC5AC (human) | Forward: 5’- AGCCGGGAACCTACTACTCG -3’ |
|  | Reverse: 5’- AAGTGGTCATAGGCTTCGTGC -3’ |
| β-actin(human) | Forward: 5’- TTGCAGCTCCTTCGTTGCC -3’ |
|  | Reverse: 5’- GACCCATTCCCACCATCACA -3’ |
| U6 (human) | Forward: 5’- CTCGCTTCGGCAGCACA -3’ |
|  | Reverse: 5’- AACGCTTCACGAATTTGCGT -3’ |
| hsa-miR-34b  hsa-miR-34c | Forward:5’-ACGGGCTAGGCAGTGTCATTAGCTGATTG -3’ |
|  | Reverse: 5’- CGCGAGGCAGTGTAGTTAGCTGATTGC -3’ |
